# Hearing Performance in Cochlear Implant Users Who Have Facial Nerve Stimulation *

**DOI:** 10.1055/s-0043-1775809

**Published:** 2023-11-14

**Authors:** Larissa Veloso Rocha, Maria Valéria Schmidt Goffi-Gomez, Ana Cristina Hoshino, Robinson Koji Tsuji, Ricardo Ferreira Bento

**Affiliations:** 1 Department of Otorhinolaryngology, Hospital das Clínicas, Faculty of Medicine, Universidade de São Paulo, São Paulo, SP, Brazil

**Keywords:** hearing loss, cochlear implantation, rehabilitation, facial nerve

## Abstract

**Introduction** Facial nerve stimulation (FNS) is a complication in cochlear implant (CI) when the electrical current escapes from the cochlea to the nearby facial nerve. Different management to reduce its effects are available, although changes might result in a less-than-ideal fitting for the CI user, eventually reducing speech perception.

**Objective** To verify the etiologies that cause FNS, to identify strategies in managing FNS, and to evaluate speech recognition in patients who present FNS.

**Methods** Retrospective study approved by the Ethical Board of the Institution. From the files of a CI group, patients who were identified with FNS either during surgery or at any time postoperatively were selected. Data collection included: CI manufacturer, electrode array type, age at implantation, etiology of hearing loss, FNS identification date, number of electrodes that generated FNS, FNS management actions, and speech recognition in quiet and in noise.

**Results** Data were collected from 7 children and 25 adults. Etiologies that cause FNS were cochlear malformation, head trauma, meningitis, and otosclerosis; the main actions included decrease in the stimulation levels followed by the deactivation of electrodes. Average speech recognition in quiet before FNS was 86% and 80% after in patients who were able to accomplish the test. However, there was great variability, ranging from 0% in quiet to 90% of speech recognition in noise.

**Conclusion** Etiologies that cause FNS are related to cochlear morphology alterations. Facial nerve stimulation can be solved using speech processor programming parameters; however, it is not possible to predict outcomes, since results depend on other variables.

## Introduction

Facial nerve stimulation (FNS) is a complication in cochlear implant (CI) programming. The electrical current, passing through the electrodes to the spiral ganglion cell, may spread to the nearby facial nerve. There are varying degrees of FNS experienced by CI users, ranging from mild stimulation or slight movement in the eye, mouth, nasolabial rhyme or forehead regions to global stimulation and severe gross movement of the total facial musculature and/or severe pain. 1

Facial nerve stimulation may appear during surgery, be observed immediately after CI activation, or up to 10 years after regular use. 2 The onset might be difficult to determine because it might progress over time or develop as a result of a new fitting (map). The diagnosis of FNS often relies on self-reporting, which can be difficult for the pediatric population in whom FNS often remains unidentified, misdiagnosed or unreported, 3 until it may be identified by the audiologist in routine fitting sessions.

Possible explanations of a FNS include the proximity of the facial nerve to the outer wall of the cochlea; the use of high rates of electrical current to stimulate the auditory nerve, as in cases of hypoplastic acoustic nerves; decreased otic capsule impedance, as in cases of otosclerosis or postmeningitis; decreased impedance at the modiolar base, as in cases of temporal bone fracture; and leakage currents due to a change in bone properties, as in cases of otosclerosis. 4 5

Some actions may be used to reduce the effects of this stimulation, such as deactivation the electrodes that cause FNS, adjusting current levels, or using triphasic pulses instead of biphasic pulses, and changing the coding strategy from monopolar to bipolar stimulation. 6 7 These changes may result in less-than-ideal maps for the experienced CI user, with a possible reduction in auditory perception. 8

The aim of the present study was to identify the etiologies that may lead to facial nerve stimulation in cochlear implant users; to identify the most common strategies in managing the problem; and to evaluate speech recognition in quiet and in noise of patients who present FNS.

## Material and Methods

This is a retrospective and descriptive study based on data from medical records of patients from the Cochlear Implant Group of this institution.

After approval of the project by the Research Ethics Committee of this institution (CAAE: 55481922.0.0000.0068), the medical records of all patients were selected according to the established criteria.

### Inclusion Criteria

- Users of CIs who referred or who presented stimulation of the facial nerve in the speech processor programming.

### Exclusion Criteria

- Patients who have peripheral facial palsy on the cochlear implant side.

Studied variables were presented with descriptive methods, by measures of central tendency and dispersion in each CI manufacturer.

The following variables were collected:

- Electrode array model (straight or perimodiolar and number of electrodes from the company);
- Age at implantation;
- Etiology of hearing loss;
- Facial nerve stimulation identification time (intraoperatively, at activation or months after activation);
- Identification of the FNS (reported or observed);
- Actions taken to resolve the FNS;
- Speech recognition in quiet before and after FNS and in noise before and after FNS at the last fitting appointment;
- Number of active electrodes (channels) on the map in use;
- Stimulation mode on map in use.

## Results

Our group had 2,082 CI ears by the time of the study. Facial nerve stimulation was identified in 32 cochlear implant users, 7 children, with a mean age of 3 years and 7 months and 25 adults with a mean age of 43 years and 8 months.

Etiologies found to cause FNS were mainly due to cochlear morphology alterations such as cochlear malformation, head trauma, meningitis, otosclerosis, or after cancer treatment such as radiotherapy ( Table 1 ).

**Table 1:** Analysis of the studied variables by CI manufacturer

CI ears implanted in our group	AB Total = 229	Cochlear Total = 1099	MED EL Total = 475	OM Total = 279
	AB *n* = 3	Cochlear *n* = 12	MED EL *n* = 15	OM *n* = 2
**Electrode array type**				
** Straight**	0	12	15	2
** Pre curved**	3	0	0	0
**Etiology**				
** Unknown**	2	1	3	−
** Meningitis**	1	1	4	−
** Otosclerosis**	−	3	1	−
** Cochlear Malformation**	−	1	5	−
** Hypoplasia**	−	−	1	−
** Rubella**	−	−	1	−
** Trauma**	−	5	0	−
** Genetics**	−	1	−	−
** Radiotherapy/Chemotherapy**	−	−	−	1
** Otitis media**	−	−	−	1
**FNS**				
** Observed**	2	10	11	2
** Reported by the patient**	1	2	4	0
**Average number of electrodes that cause FNS (min – max)**	1 (NID)	7 (1–22)	4 (1–12)	15 (12–18)
**Time of FNS identification**				
** Intraoperative/activaction**	0	9	5	2
** Months postoperatively (min-max)**	3 (3–26)	3 (8–32)	10 (1–74)	0

Abbreviations: CI, cochlear implant; FNS, facial nerve stimulation; NID, not identified or no details found in the patient́s file.

Among the actions performed, the decrease in the electric current followed by the deactivation of the electrodes that generated this stimulation were identified as the most common ( Table 2 ). In some cases, when FNS was identified during intraoperative assessment of the neural response, no action was required since it did not appear with the parameters of the fitting map.

**Table 2:** Description of the fitting management of the FNS by CI manufacturer

	AB *n* = 3	Cochlear *n* = 12	MED EL *n* = 15	OM *n* = 2
**Managing action**				
** No action required**	0	3	0	1
** Decrease of stimulation levels**	3	4	9	1
** Pulse width increase**	0	1	0	0
** Change of stimulation mode**	0	0	0	0
** Pulse change (from bipolar to tripolar)**	0	0	3	0
** Electrode deactivation**	0	4	2	0
** NID**	0	1	0	0
** Electrode array reposition (intraop)**	0	0	1	0
**Average number of deactivated electrodes (min – max)**	2 (0–3)	6 (1 - 16)	2 (0–6)	1 (0 - 2)

Abbreviations: CI, cochlear implant; FNS, facial nerve stimulation; NID, not identified or no details found in the patient́s file.

Not all patients could be tested in all tasks, since some have 0% speech recognition in quiet (open set presentation). Table 3 displays the average performance as well as the number of patients that were assessed in each task. The average speech recognition in patients who accomplish open set presentation was 86% before identification of the FNS and 80% after fitting management. However, there was great variability, from 30% in closed presentation to 90% recognition in noise ( Table 3 ).

**Table 3:** Speech recognition in quiet and in noise before and after FNS resolution by CI manufacturer

	AB	Cochlear	MED EL	OM
**Speech recognition in quiet (open set)**				
*** n***	2	7	5	0
** % (min - max)**	90% (80–100)	81,4% (30–100)	74% (50–100)	−
**Speech recognition in quiet (closed set)**				
*** n***	1	1	0	1
** % (min - max)**	50%	30%	−	50%
**Speech recognition in noise**				
*** n***	2	6	4	0
** % (min - max)**	10% (10–10)	41,6% (10–80)	47,5% (10–80)	−

Abbreviations: CI, cochlear implant; FNS, facial nerve stimulation.

In Graphs 1 and 2 , we analyze task performance in more detail, looking at speech recognition before FNS in silence in open set and closed set (for those who had postoperative FNS, that is, 9 patients in all) and after changes have been made to reduce this extra auditory stimulation. The graphs show that, in this sample, 44.4% of the patients improved their speech recognition performance after the new programming, 33.3% maintained their performance, and 22.2% showed a deterioration in their performance.

**Graphic 1:**
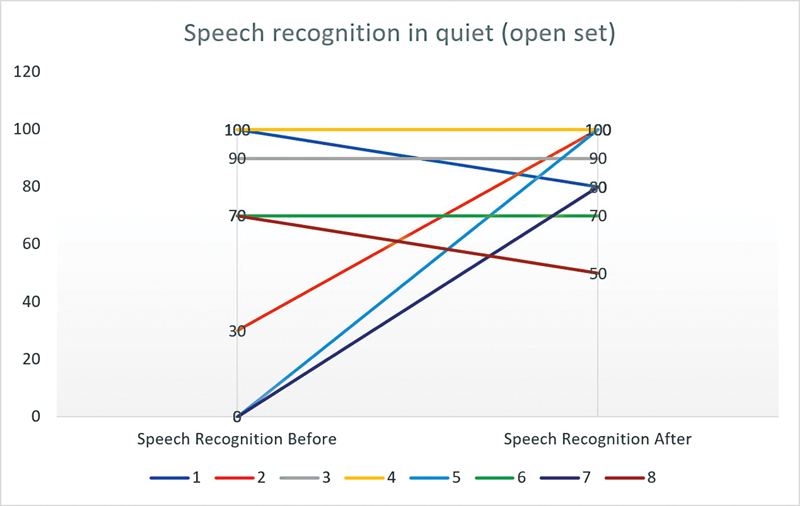
Speech recognition in quiet (open set) before and after FNS resolution by CI manufacturer.

**Graphic 2:**
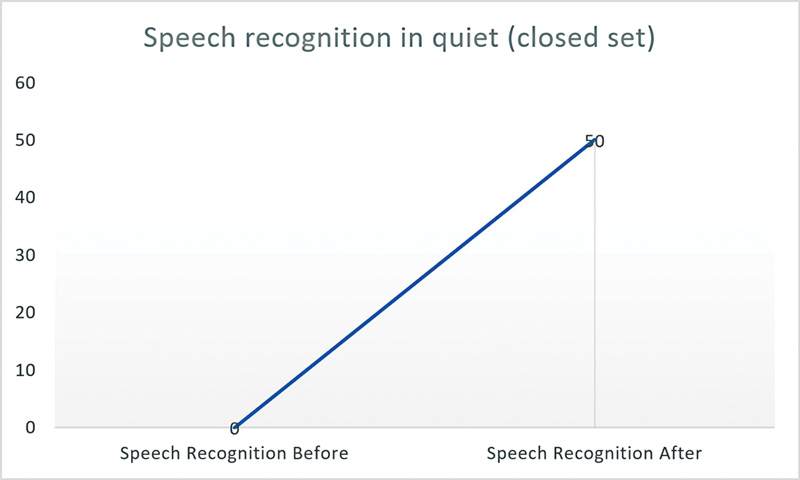
Speech recognition in quiet (closed set) before and after FNS resolution by CI manufacturer.

## Discussion

The present study was designed with the purpose of performing a descriptive analysis of the selected population so that, in the future, it would be possible to create preventive measures for facial nerve stimulation (FNS) after cochlear implant surgery.

Data were collected from 32 cochlear implant users, among children and adults, users from the four main cochlear implant companies (Advanced Bionics, Cochlear Corporation, Med-EL, and Oticon Medical).

Most patients in this sample were users of the Med-El CI (46.8%), probably due to the company's extensive portfolio of electrodes, representing a choice for cases of cochlear malformation, which is one of the etiologies with the highest occurrence in this study.

The electrode array was straight in 90.6% of the cases with FNS. Previous studies have reported that the electrode design may influence the incidence of FNS. It was demonstrated that 43% of CI users with straight arrays presented with FNS, while none of those with perimodiolar electrodes presented with FNS. 9 Frijns et al. reported that perimodiolar arrays with more focused electrical stimulation (and therefore lower stimulation levels and less current spread) may decrease the likelihood of FNS. 10

Two other studies (6 cases of CI in total) of FNS have described patients with unsatisfactory audiological results, these had the straight electrode device replaced by a perimodiolar one and reported great improvement in the audiological outcomes and no FNS. 11 12 This fact raises the hypothesis that not only the length of the electrode array, but also its design may reduce the cases of FNS in CI users, considering most of the recent precurved electrode arrays have half-band electrodes.

Patients in this sample were diagnosed with different etiologies, such as cochlear malformation (18.7%), head trauma (15.6%), meningitis (18.7%), and otosclerosis (12.5%). Despite these findings having already been mentioned in the literature, 4 5 other less common etiologies lead to FNS in our sample, such as a case of head and neck cancer, who underwent treatment with radiotherapy and chemotherapy. Those treatment approaches may have led to a more fragile temporal bone, due to radiation-induced injuries, 13 allowing the leak of electric current from the cochlea, generating the FNS.

In 78.1% of the cases, the FNS was observed by the audiologist, and in 21.8% of the cases, the patient reported the nonauditory sensation. Cushing et al. suggested that children may not be able to comment on the presence of facial spasms and, therefore, it is possible that cases of FNS remain undetected in this age group. 3 Therefore, it is extremely important that the audiologists keep a detailed and careful observation of the face of the child while programming the cochlear implant so that it is possible to perceive when stimulation of the facial nerve is taking place.

In half of the analyzed cases, FNS was identified intraoperatively or upon activation of the speech processor. In the other 50% of cases, a large variation was observed in the FNS start date in the months following activation. This variation is present in three of the four main CI companies starting from the first month of follow-up until after 74 months of continuous use of the CI, as observed in the literature. 2 14 In a study by Smullen et al., it was reported that the onset of FNS occurs in most patients within the 1 ^st^ year after implantation, but can develop up to 10 years later. The researchers concluded that this could be due to a change in the current pathway, a change in tissue impedance, or a sensitivity of the facial nerve. 8

We observed that there is no expected average of electrodes that can stimulate and/or that will need to be deactivated, as there is a large variation; what is known is that when many electrodes require deactivation, the performance of the device can be negatively impacted, 15 consequently, the performance of the CI user will be lower than expected.

When FNS is identified in the programming of the speech processor, the speech therapist can make a series of decisions to prevent extra auditory stimulation and maintain sound quality and auditory benefits for users. The main initial actions performed in this sample were the decrease in the electrical current, followed by the deactivation of the electrodes that generated this stimulation.

Other initial actions were observed in the individuality of each IC brand. In the CIs manufactured by Med-El, there was a change in the pulse type from biphasic to triphasic. This resource, available only in this brand of Cis, was previously studied and presented good results; the triphasic pulse is capable of reducing the effects of FNS, distributing the charge over two negative phases of the same duration and a positive phase with a double duration. 16 Meanwhile, at Cochlear Corporation, a change in the stimulation mode was observed, changing the mode from monopolar to bipolar, allowing a more restricted electric field, dispersing the electric current less, and being able to avoid and/or decrease FNS. 17

In our study, data regarding the recognition of sentences in different conditions were collected. The average of speech recognition in open presentation before the identification of the FNS was 57,5% of correct answers; however, after the identification of the FNS and adjustments had been made to avoid extra hearing aid stimulation, the average speech recognition was 83,7% correct. However, a great variability was observed in the results for each patient, from 30% of correct answers in closed presentation to 90% of correct answers in recognition in noise.

The main variables that may have interfered were etiology, number of deactivated electrodes and maximum level of electrical current. However, it is extremely important that the professional responsible for programming the speech processor is aware that these changes, such as the deactivation of electrodes and the decrease in electrical current, despite reducing the FNS, can impact the user's auditory abilities, since the map will be below adequate. Braun et al., in a 2019 study, demonstrated that in pure tone audiometry and speech tests, there were no consequences after adjustments were made to decrease or cease FNS; the patients studied continued with good results in the tests, being able to speak on the phone with a known person. 16 Alzhrani et al. also observed good auditory performance in this population even after FNS reduction. 18

## Conclusion

Etiologies that cause FNS are related to cochlear morphology alterations.

Facial nerve stimulation may be solved using speech processor programming parameters. Nevertheless, it is not possible to predict the auditory outcomes of patients who will have FNS, as the results depend on other variables inherent to each implanted patient.
